# Human visceral leishmaniasis and relationship with vector and canine control measures

**DOI:** 10.11606/S1518-8787.2018052000381

**Published:** 2018-11-14

**Authors:** Danielle Nunes Carneiro Castro Costa, Patricia Marques Moralejo Bermudi, Lilian Aparecida Colebrusco Rodas, Caris Maroni Nunes, Roberto Mitsuyoshi Hiramoto, José Eduardo Tolezano, Rafael Silva Cipriano, Graziela Cândido Diniz Cardoso, Cláudia Torres Codeço, Francisco Chiaravalloti

**Affiliations:** I Universidade de São Paulo. Faculdade de Saúde Pública. Programa de Pós-Graduação em Saúde Pública. São Paulo, SP, Brasil; II Superintendência de Controle de Endemias. Serviço Regional 9. Araçatuba, SP, Brasil; III Universidade Estadual Paulista. Faculdade de Medicina Veterinária de Araçatuba. Departamento de Apoio Produção e Saúde Animal. Laboratório de Bioquímica e Biologia Molecular. Araçatuba, SP, Brasil; IV Instituto Adolfo Lutz. Núcleo de Parasitoses Sistêmicas. São Paulo, SP, Brasil; V Prefeitura Municipal de Araçatuba. Secretaria Municipal de Saúde. Centro de Controle de Zoonoses. Araçatuba, SP, Brasil; VI Fundação Oswaldo Cruz. Programa de Computação Científica. Rio de Janeiro, RJ, Brasil; VII Universidade de São Paulo. Faculdade de Saúde Pública. Departamento de Epidemiologia. São Paulo, SP, Brasil

**Keywords:** Leishmaniasis, Visceral, prevention & control, Dogs, parasitology, Euthanasia, Animal, Spatial Analysis, Leishmaniose Visceral, prevenção & controle, Cães, parasitologia, Eutanásia Animal, Análise Espacial

## Abstract

**OBJECTIVE:**

Estimate the coverage of control measures of visceral leishmaniasis and relate them with the occurrence of human visceral leishmaniasis in endemic urban area.

**METHODS:**

Cases of human and canine visceral leishmaniasis were considered as study population and evaluated by a serological survey conducted in Araçatuba, state São Paulo, from 2007 to 2015. The cases of human visceral leishmaniasis were geocoded by the address of the patients and the canine disease by the address of the dogs’ owners. The coverage of serological survey, euthanasia, and insecticide spraying was calculated, as well as the canine seroprevalence and the incidence rates of human visceral leishmaniasis. The relationship between human visceral leishmaniasis and control measures was evaluated, as well as the seroprevalence by comparing maps and by linear regression. The relationship between the canine and the human disease was also evaluated by the Ripley’s K function.

**RESULTS:**

The incidence rates of human visceral leishmaniasis showed a period of decline (2007 to 2009) and a period of stability (2010 to 2015), a behavior similar to that of canine seroprevalence. In general, the coverage of control measures was low, and the non-association with the incidence of human visceral leishmaniasis can be a result of the period analyzed and of the small number of analyzed units (sectors of the Superintendence for the Control of Endemic Diseases). The distribution of human cases showed spatial dependence with the distribution of seropositive dogs from 2007 to 2009.

**CONCLUSIONS:**

This study reaffirmed the relationship between the occurrence of the disease in humans and dogs, it verified a decrease in the rates of visceral leishmaniasis in Araçatuba over time, even at low coverage of control activities. However, further studies are needed to determine if factors beyond monitoring and control measures are involved in the reduction of incidences.

## RESUMO

**OBJETIVO:**

Estimar a cobertura das atividades de controle da leishmaniose visceral e relacioná-las com a ocorrência de leishmaniose visceral em humanos em área urbana endêmica.

**MÉTODOS:**

Foram considerados como população de estudo os casos de leishmaniose visceral em humanos e em cães avaliados por inquérito sorológico censitário realizado em Araçatuba, SP, de 2007 a 2015. Os casos de leishmaniose visceral em humanos foram geocodificados pelo endereço de residência dos pacientes e, os cães, pelo endereço de residências dos respectivos tutores. Foram calculadas as coberturas do inquérito sorológico, da eutanásia e de borrifação de inseticida, as soroprevalências caninas e as taxas de incidência de leishmaniose visceral em humanos. A relação entre a leishmaniose visceral em humanos e as medidas de controle, bem como a soroprevalência foram avaliadas por comparação de mapas e por meio de regressão linear. A relação entre a doença canina e a humana também foi avaliada por meio da função *K de Ripley* .

**RESULTADOS:**

As taxas de incidência de leishmaniose visceral em humanos apresentaram um período de declínio (2007 a 2009) e um período de estabilidade (2010 a 2015), comportamento semelhante ao das soroprevalências caninas. Em geral, a cobertura das medidas de controle foi baixa e a não associação com a incidência de leishmaniose visceral em humanos pode ser consequência do período analisado e do número pequeno de unidades analisadas (setores da Superintendência de Controle de Endemias). A distribuição dos casos humanos apresentou dependência espacial com a distribuição dos cães soropositivos de 2007 a 2009.

**CONCLUSÕES:**

Este trabalho reafirmou a relação entre a ocorrência da doença no homem e no cão, verificou a diminuição das taxas de leishmaniose visceral em humanos e em cães em Araçatuba ao longo do tempo, mesmo em baixa cobertura das atividades de controle. Entretanto, novos estudos são necessários para averiguar se fatores além das atividades de vigilância e controle estariam envolvidos na diminuição das incidências.

## INTRODUCTION

Visceral leishmaniasis is one of the six priority endemic diseases in the world. It is a neglected tropical disease that, if left untreated, can present high lethality in humans. In addition, this disease evolves in malnourished individuals or carriers of the human immunodeficiency virus. In Brazil, this zoonosis was considered typical of rural areas with about 90% of the reported cases in the Northeast region^1 – 3^ . Visceral leishmaniasis (VL) has been expanding to other Brazilian regions since the 1980s, especially for the Southeast region. This expansion is related to the processes of urbanization, deforestation and human migration, among other factors ^4^ .

The measures of the Visceral Leishmaniasis Monitoring and Control Programme (VLMCP) are directed to the host, by education and healthcare activities and treatment of human cases; to the vector, by entomological research on phlebotomine transmitting VL, insecticide spraying in intra- and peridomiciliary areas and environmental management; and to the canine reservoir, by the canine population control and by euthanasia of seropositive dogs. However, we wonder if these actions are effective enough so that the incidence of the disease is reduced in Brazil^5–7^. The Brazilian Ministry of Health (MS) accepts the vaccination of dogs against canine visceral leishmaniasis (CVL) and the use of deltamethrin-impregnated collars as individual control activities, since the effectiveness of these actions as public health control measures were not proven^1–3^.

This study aimed to measure the coverage of the activities of chemical control and of the canine reservoir, relating them to the occurrence of human cases in Araçatuba, state São Paulo. This municipality was chosen as study area because of its epidemiological importance. Araçatuba was the first city to verify the presence of the vector in the state of São Paulo, in 1997, and the first city to confirm an indigenous case of the disease in humans, in 1999, a year after the registration of canine cases ^5^ ^,^ ^8^ . Since then, the urban area of the city has become endemic for the disease, although a decrease in the number of human cases has been observed lately ^9^ .

## METHODS

The study was carried out in the urban area of Araçatuba, located in the Northwest region of the state of São Paulo, endemic for VL since 1999, with a population estimated at 193,828 inhabitants ^10^ . Cases of human and canine visceral leishmaniasis were considered as study population and evaluated by a serological survey conducted in Zoonoses Control Center of Araçatuba (CCZ), state São Paulo, from 2007 to 2015.

The sectors delimited and used by the city and by the Superintendence for the Control of Endemic Diseases (SUCEN) to carry out monitoring and control activities, defined as “SUCEN sectors,” were established as units of analysis. These units were composed of two or more census tracts, elaborated digitally using the dissolve operation (for matching areas) and the node tool (for unmatched areas) of the QGIS program, version 2.16.2 ^11^ . To do so, a physical map of Araçatuba provided by the municipality, containing the delimitations of these sectors and census sectors, was used, as well as a digital map of the census sectors, obtained from the Brazilian Institute of Geography and Statistics (IBGE). In addition to the construction of the digital map, the information on segregated populations in census sectors was dissolved in accordance with the “SUCEN sectors”.

Among monitoring and control activities of VL, the chemical control in properties in the surrounding areas of indigenous cases of HVL was considered for the elimination of the vector (insecticide spraying in intra- and peridomiciliary areas), and the identification of seropositive dogs by serologic tests and euthanasia (canine reservoir control). This information, registered in field bulletins that were made available by CCZ, was included in electronic spreadsheets. The recommendations contained in the Manual on Visceral Leishmaniasis Monitoring and Control of MS and of the state of São Paulo were used as references for the evaluation of the coverage of these control activities^1–3^.

The coverage of activities of insecticide spraying was calculated by the ratio of the number of properties sprayed and the number of properties programed for this purpose, i.e. the ratio held by the scheduled. An area of at least 200 m radius was considered around the location of the cases registered of HVL in the previous two years for the calculation of properties that should be sprayed in a certain year ^1^ .

The annual census serological canine survey was planned for regions with intense transmission of the disease until 2016. Once Araçatuba is in this classification of risk, the serological survey coverage was calculated by the division between the number of dogs evaluated by CCZ and the number of existing dogs, taking as a basis the proportion of one dog per five people, for each year studied ^12^ . The dog is considered seropositive if it shows positive results in both diagnostic tests used (a screening and a confirmatory test). Seroprevalence was calculated by dividing the number of seropositive dogs by the number of dogs evaluated in the same period. As for euthanasia, its coverage was calculated by dividing the number of dogs put down by the number of seropositive dogs.

The information about the HVL cases (date of onset of symptoms, age and address) was obtained based on the information recorded in the Notifiable Diseases Information System (SINAN), provided by the Municipal Health Secretariat of Araçatuba. Incidence rates were calculated (by area and time period) and presented by age groups of 0–19 years, 20–59 years and 60 years or more, and the percentage of reduction of the incidence rate was calculated from 2007 to 2009 and from 2010 to 2015 ^10^ .

The HVL cases of human visceral leishmaniasis were geocoded by the address of the patients and the dogs evaluated in the survey by the address of their respective owners. Before the geocoding, the standardization of data addresses in the format of the addresses on the street map of Araçatuba was necessary in order to identify the public parks through the TerraView ^13^ program. The coverage of insecticide spraying, serological survey and euthanasia, the canine seroprevalence, and HVL incidence rates were calculated by year and age groups, according to the “SUCEN sectors” for the whole urban area of the municipality and presented in tables and maps elaborated in the program QGIS, version 2.16.2 ^11^ .

The relationship between human and canine disease and control measures were evaluated based on the comparison of the respective maps obtained for each year of the study period. This assessment was also carried out with the use of linear regression models. For this analysis, considering the temporal behavior of the incidence rate, the study period was divided into two subperiods (2007 to 2009 and 2010 to 2015), and a model was created for each subperiod. For modeling, the dependent variable was the incidence rate, and the independent variables was the coverage of insecticide spraying, serological survey, euthanasia and canine seroprevalence.

The exploratory analysis of the data was performed before modeling ^14^ . For the multiple linear regressions models, the method adopted to choose the most suitable models was the method based on likelihood ratio, considering as the best-fit criterion the models that presented lower Akaike’s Information Criterion (AIC) ^15^ . These analyses were performed on the program *R,* version 3.2.3.

To assess the existence of association in the space between the occurrence of the disease in humans and dogs, bivariate analyses were made by the Ripley’s K function (for both subperiods) ^16^ . It is a technique of analysis of points that evaluated the hypothesis of the existence of spatial dependence between specific distributions of HVL and CVL cases.

This study received the approval of the Research Ethics Committee of the Faculdade de Saúde Pública at the Universidade de São Paulo (CAAE 38170514.4.0000.5421 – Opinion 892,518 – date of Rapporteur: Dec 11, 2014).

## RESULTS

**Figure 1:**
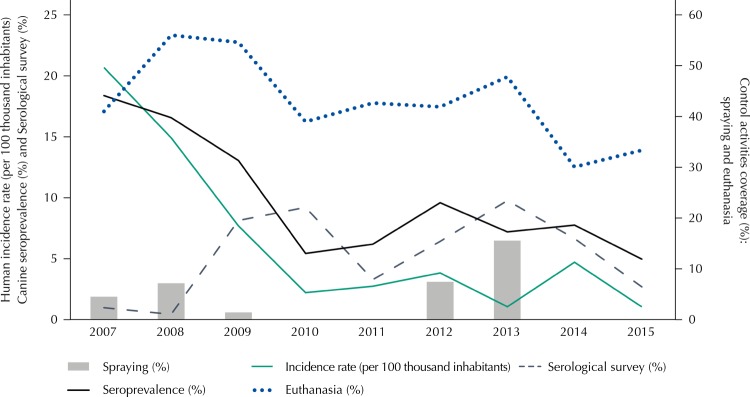
Incidence rates for human visceral leishmaniasis (VL) (per 100 thousand inhabitants), canine seropositivity (%), and coverage of control activities of VL: insecticides spraying (%), serological survey (%), and euthanasia (%), according to the study year. Aracatuba, state of São Paulo, 2007 to 2015.

**Figure 2:**
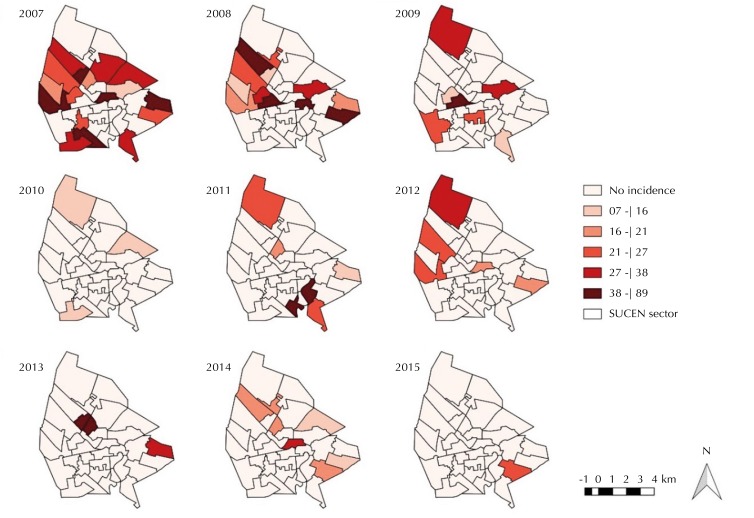
Maps of the distributions of incidence rates of HVL (per 100 thousand inhabitants) according to the “SUCEN sector.” Aracatuba, state of São Paulo, 2007 to 2015.

HVL: human visceral leishmaniasis; SUCEN: Superintendence for the Control of Endemic Diseases

The curve of the temporal trend of the canine seropositivity and of the incidence rate of HVL in the urban area of Araçatuba, between 2007 and 2015 ( Figure 1 ), presented patterns that can be divided into two distinct periods: a period of decline, between 2007 and 2009, followed by a period of apparent stability, between 2010 and 2015, in which the average seroprevalence was 6.8%, with values between 5.0% and 10.0%. The average incidence rate was 2.6 cases per 100 thousand inhabitants, with an average of 4.8 cases/year. The percentage of reduction of incidence rate between periods was 79.0%, and a similarity was observed in the percentage by age group: 79.0% in the group from zero to 19 years, 78.0% from 20 to 59 years, and 81.0% from 60 years or more. The decrease in rates in the whole urban area was followed by the decrease over time, in the number of “SUCEN sectors” with HVL cases and in the magnitude of rates in these sectors ( Figure 2 ).

The canine serological survey showed coverage between 1.0% and 10.0%, with the lowest value in 2008 and the highest in 2013. It did not provide any temporal relationship with the HVL incidence rate, nor with canine seroprevalence. Euthanasia coverage ranged from 30.0% and 60.0% and presented its highest value in 2008, which was followed by decrease, in accordance with the decrease in seroprevalence. Insecticide spraying was the control measure that presented the lowest coverage with values below 20.0% and null coverage in four of the nine years of the study ( Figure 1 ).

**Figure 3:**
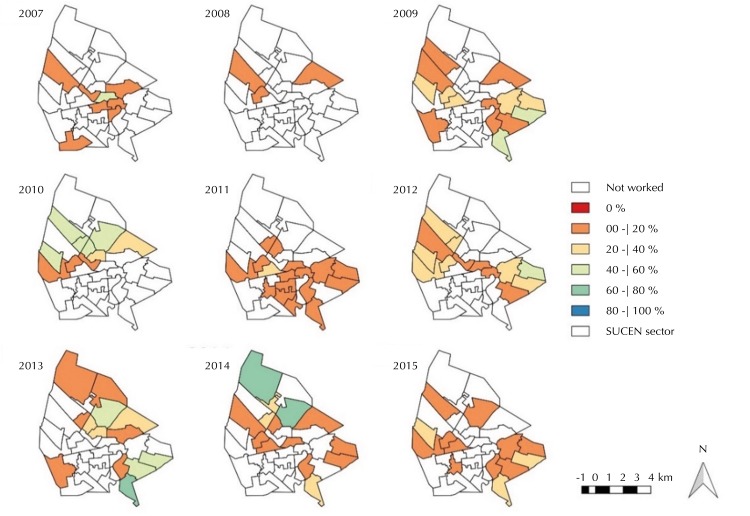
Maps of the distributions of the coverage of the canine serological survey, according to the “SUCEN sector.” Urban area of Araçatuba, state of São Paulo, 2007 to 2015.

The survey coverage, according to the years and to the “SUCEN sectors,” in general, was lower than 40.0% and null in many sectors. The years 2007 and 2008 showed the smallest coverage and the highest numbers of “SUCEN sectors” not worked ( Figure 3 ). Comparing Figures 2 and 3 , we observe that the implementation of canine serologic surveys was frequent in areas with human cases in the same year and in the subsequent two years.

SUCEN: Superintendence for the Control of Endemic Diseases

**Figure 4:**
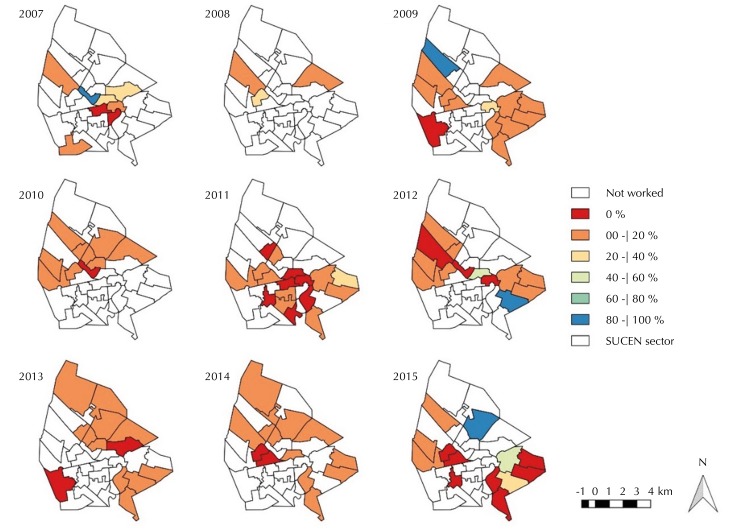
Maps of the distributions of canine seropositivity for VL, according to the “SUCEN sector.” Urban area of Araçatuba, state of São Paulo, 2007 to 2015.

SUCEN: Superintendence for the Control of Endemic Diseases; VL: visceral leishmaniasis

The CVL, measured only in evaluated “SUCEN sectors,” presented prevalence between 0.0% and 40.0% ( Figure 4 ). The sectors with null or seroprevalence over 40.0% showed serologic survey coverage, in almost its entirety, less than or equal to 20.0% ( Figure 3 ). Most sectors with seropositive dogs performed euthanasia coverage between 40.0% and 100.0%, but there were sectors with less than 40.0% and several others with null coverage.

Based on the identification of two periods with different behaviors in the temporal distribution of the incidence rate of HVL ( Figure 1 ), we decided to perform its modeling according to these two periods: 2007 to 2009 (decline) and 2010 to 2015 (stability). The exploratory analysis, carried out in accordance with these two periods, showed the need for exclusion of insecticide spraying coverage, due to the small number of worked “SUCEN sectors” and of the transformation of variables to work around problems of outliers in the covariates. The analysis of collinearity between the covariates showed the need to eliminate euthanasia coverage in the first period.

The linear regression model of the incidence rate of the first period showed that the smaller AIC model was that with an intercept equal to 3.20 and significant intercept (p < 0.001), and with variable serological survey without statistical significance (coefficient equal to 00.7; p = 0.140). In the second period, the best model included intercept equal to 2.57 (p = 0.010) and none of the covariates. The waste of these models showed normal distribution, homoscedasticity and absence of spatial autocorrelation.

No significant associations were observed between the rates of HVL and the canine seroprevalence and coverage of the control activities in both study periods. The positive relationship, although not significant, between the incidence rates and coverage of the serological survey in the first study period agreed with the observation made above after comparing Figures 2 and 3: the second activity would follow the first regarding space, but with some time lag.

**Figure 5:**
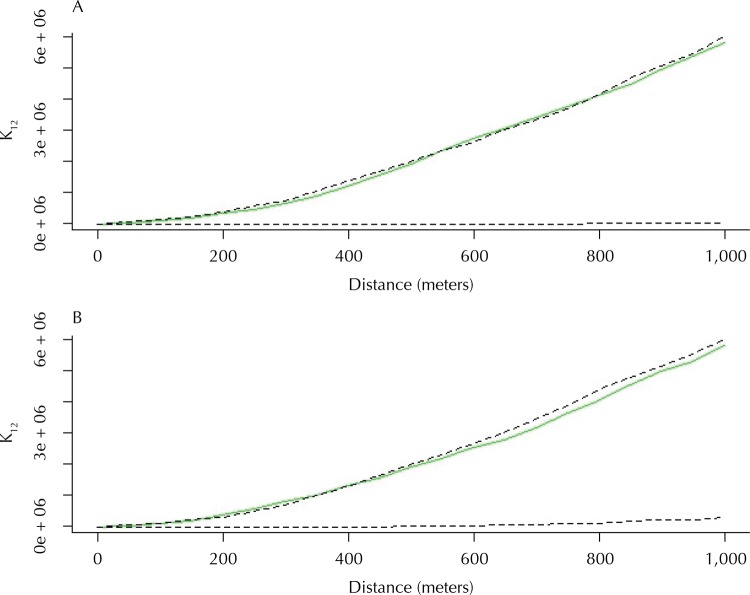
Charts of distributions of spatial dependency between human visceral leishmaniasis in relation to the canine disease, according to bivariate analysis by the Ripley’s K function. Araçatuba, state São Paulo, 2007 to 2009 (A) and 2010 to 2015 (B).

Spatial dependence (or attraction) was identified, at distances of 400 m to 800 m, between the distribution of points of HVL cases and seropositive dogs in the first study period ( Figure 5 , A). These two variables showed spatial dependence, which means that the human cases and seropositive dogs were closer than would be expected if the distributions were random. With the reduction of the force of VL infection, no statistically significant spatial dependence was identified between the two variables from 2010 to 2015. The default aggregation of data and trust boundaries were similar in both curves; however, the limited number of samples in the second period (2010 to 2015) may have hampered the observation of spatial relationship ( Figure 5 , B).

## DISCUSSION

VL control measures recommended by VLMCP are based on the restoration of the patient’s health, on reducing the density of the vector and the sources of canine infection, aiming to decrease the transmission potential to humans. Epidemiological studies found overlap between locations with high incidence of human cases and canine seroprevalence in urban areas, showing the close relationship between the human and the canine disease^4 , 17 – 19^ . Similarly, in this study, spatial dependence was observed in human and canine cases, at least from 2007 to 2009. In addition, there was an agreement in the time between the occurrence of the disease in humans and in dogs throughout the study period.

Control measures (insecticide spraying and control of canine reservoir) showed, in general, low coverage in the study period. Spraying had the lowest coverage, which prevented the evaluation of the effectiveness of this measure in reducing the incidence of HVL. The difficulties in the vector control go beyond the vector adaptability to urban environments and different temperatures. This contributes to the dispersion of areas free of disease. The complexity of chemical control and its several operational difficulties, such as the high refusal to apply the insecticide in the intra- and peridomiciliary areas and, mainly, the lack of human and material resources may have contributed to the low coverage ^7^ ^,^ ^20^ ^,^ ^21^ . However, this is an important strategy for the VL control, since it can contribute to the reduction of the number of bites that result in the transmission of the infectious agent, hindering the transmission of the disease^1–3,6^.

The focus of canine control actions in Araçatuba were the areas with HVL to reduce the transmission potential of these affected areas. However, the non-implementation of monitoring and control activities in regions without human cases may cooperate with the circulation of the disease, since the canine disease precedes the human disease ^22^ ^,^ ^23^ .

Thus, given the low coverage of serologic surveys, seroprevalence must be examined with caution, as it may not represent the actual prevalence of CVL throughout the municipality. Seroprevalence is influenced by the number of dogs subjected to diagnostic tests in the areas sampled. The fact that sampling is not probabilistic and that the number of units of analysis is limited may have generated a bias regarding the seroprevalence analysis. The proportion of infected dogs that stopped being sampled compared with healthy dogs is unknown, as well as whether areas with greater or lesser seroprevalence were sampled with different sampling fractions. However, the reduction of seroprevalence can cooperate with the decrease in incidence rates of HVL, because the appearance of human cases is estimated to occur after two years of seroprevalence levels of CVL over 20% ^23^ .

Similarly, the high euthanasia coverage can be a consequence of the low coverage of serological surveys. Only the dogs who participated in the surveys could be assessed and the seropositive dogs put down. Therefore, the low coverage of the serological survey allows the permanence of infected dogs in the environment, ensuring the transmission dynamics of the disease.

The high cost of VLMCP actions, which do not always consider the local reality, leads to discontinuity of these activities, especially during epidemics of dengue fever, since the VL control, in general, uses the same human and financial resources that are administered for the control of other diseases in force in the municipality. This competition for the resources brings prejudice to that disease with less popular appeal, in this case, to VL ^21^ . In addition, the low effectiveness of the canine reservoir control is related to the permanence of seropositive dogs in the environment. This occurs by several factors, such as: dogs that are not evaluated; low coverage of serological surveys; issues related to the characteristics of the diagnostic tests, which may not detect infected dogs during the incubation period of the disease, resulting in false-negatives; long time between the diagnosis and euthanasia; refusal to deliver the seropositive dog by the owners; and the replacement by susceptible after compulsory euthanasia of seropositive dogs, ensuring the continuity of the transmission dynamics^4 , 6 , 8 , 17 – 21^ .

However, despite the low coverage of control measures, there was a decrease in the incidence rates of HVL and in seroprevalence of CVL, followed by a period of stability. The same pattern of occurrence of human cases was observed in the state of São Paulo ^24^ . Although the epidemiological stratification proposed by MS rates Araçatuba as an intense transmission area (areas with an average of human cases exceeding 4.4), this classification is criticized by considering the total number of cases regardless of the population size, which can mask the real transmission dynamics of HVL. In this case, the assessment, including the incidence, could be more appropriate^1 – 3 , 5^ .

One of the ways to verify the effectiveness of control measures is from the decrease in the disease prevalence among children, since this is the largest group incidence^1 – 3 , 8^ . Despite not having been observed difference by age group in the percentage of reduction of incidence rate, comparing the two study periods, the drop in the rate was significant, 79% both in the range from zero to 19 years and in the total number of individuals. This may be a sign that even at low coverage, euthanasia of seropositive dogs assists in the transmission control.

The use of secondary data and passive notification, with the likely occurrence of underreporting, are limitations of this study. Disaggregated data on control activities collected were filed in paper form, and all information was scanned in Excel spreadsheets. Data before 2007 were not found, neither with SUCEN nor with CCZ. This enabled us to verify whether the control activities in prior periods have had influence on the occurrence of human disease. Similarly, the lack of relationship between HVL, seroprevalence and control measures in the ecological model may be consequences of the unit of analysis used (“SUCEN sectors,” the same used by control program managers). The limited number of samples hindered the observation of spatial relationship between HVL and seroprevalence of CVL from 2010 to 2015. Another limitation of this study was the fact that all the control measures of the program were not analyzed, such as environmental management, the treatment of human cases, and health education.

Part of these limitations were overcome with the ecological design, the use of geographic information systems and spatial analysis, which helped the understanding of the disease transmission dynamics. Authors point out its importance in the identification of priority areas and evaluation of the effectiveness of monitoring and control measures^4 , 17 – 19 , 25 , 26^ .

Chemical control is theoretically an effective strategy against diseases transmitted by vectors. However, its effectiveness is compromised by issues related to operation and maintenance of this measure. Thus, to achieve the vector control, a set of continuous actions is needed, including periodic training of health workers, community participation, environmental management, and operational issues, which must be monitored and evaluated regularly to avoid disruption of these control measures ^6^ ^,^ ^7^ . As for the strategy of canine serological survey and euthanasia of seropositive dogs, although it sounds simple from the conceptual point of view, in practice, it involves many challenges, such as those aforementioned. However, some studies indicate the possibility of the VL control to be achieved, even at low coverage, if this strategy is carried out with continuity and regularity^27 – 29^ .

This study reaffirmed the relationship between the human and canine disease and the reduction of HVL and CVL rates, even at low coverage of control measures. The VL control seems to be based on society, no matter how small the actions taken may be. Measures, such as the development of education and health, responsible ownership, environmental management and individual prevention, as well as control measures, such as the use of insecticide-impregnated collars, vaccination and treatment of dogs, can be related to some degree of decrease in the occurrence of HVL cases ^30^ . Further studies are needed to determine the influence of these actions in the control of disease transmission.
